# Anti-Cancer Effect of Moroccan Cobra *Naja haje* Venom and Its Fractions against Hepatocellular Carcinoma in 3D Cell Culture

**DOI:** 10.3390/toxins13060402

**Published:** 2021-06-04

**Authors:** Ayoub Lafnoune, Su-Yeon Lee, Jin-Yeong Heo, Imane Gourja, Bouchra Darkaoui, Zaineb Abdelkafi-Koubaa, Fatima Chgoury, Khadija Daoudi, Salma Chakir, Rachida Cadi, Khadija Mounaji, Najet Srairi-Abid, Naziha Marrakchi, David Shum, Haeng-Ran Seo, Naoual Oukkache

**Affiliations:** 1 Laboratoire des Venins et Toxines, Département de Recherche, Institut Pasteur du Maroc, 1, Place Louis Pasteur, Casablanca 20360, Morocco; ayoublafnoune@gmail.com (A.L.); i.gourja@gmail.com (I.G.); DARKAOUI.Bouchra@hotmail.com (B.D.); fchgoury@gmail.com (F.C.); d.khadija00@gmail.com (K.D.); salmachakir19@gmail.com (S.C.); 2 Laboratoire Physiopathologie, Génétique Moléculaire & Biotechnologie, Faculté des Sciences Ain-Chock, Hassan II University of Casablanca, B.P 5366 Maarif, Casablanca 20000, Morocco; rachidacadi@gmail.com (R.C.); kh.mounaji@gmail.com (K.M.); 3 Cancer Biology Research Laboratory, Institut Pasteur Korea, 16, Daewangpangyo-ro 712 beon-gil Bundang-gu, Seong-nam-si 13488, Gyeonggi-do, Korea; suyeon.lee@ip-korea.org (S.-Y.L.); haengran.seo@ip-korea.org (H.-R.S.); 4 Screening Discovery Platform, Institut Pasteur Korea, 16, Daewangpangyo-ro 712 beon-gil Bundang-gu, Seong-nam-si 13488, Gyeonggi-do, Korea; jinyeong.heo@ip-korea.org (J.-Y.H.); david.shum@ip-korea.org (D.S.); 5 Laboratoire des Venins et Biomolécules Thérapeutiques LR11IPT08, Institut Pasteur de Tunis, 13, Place Pasteur, Tunis 1002, Tunisia; abdelkafi_zaineb@yahoo.fr (Z.A.-K.); Najet.abidSrairi@pasteur.tn (N.S.-A.); marrakchi_naziha@yahoo.fr (N.M.)

**Keywords:** anticancer molecules, *Hepatocellular carcinoma*, multicellular tumor spheroids, *Naja haje*, venom

## Abstract

Hepatocellular carcinoma (HCC) is the most common primary liver cancer in adults, the fifth most common malignancy worldwide and the third leading cause of cancer related death. An alternative to the surgical treatments and drugs, such as sorafenib, commonly used in medicine is necessary to overcome this public health problem. In this study, we determine the anticancer effect on HCC of Moroccan cobra *Naja haje* venom and its fraction obtained by gel filtration chromatography against Huh7.5 cancer cell line. Cells were grown together with WI38 human fibroblast cells, LX2 human hepatic stellate cell line, and human endothelial cells (HUVEC) in MCTS (multi-cellular tumor spheroids) models. The hepatotoxicity of venom and its fractions were also evaluated using the normal hepatocytes cell line (Fa2N-4 cells). Our results showed that an anti HCC activity of Moroccan cobra *Naja haje* venom and, more specifically, the F7 fraction of gel filtration chromatography exhibited the greatest anti-hepatocellular carcinoma effect by decreasing the size of MCTS. This effect is associated with a low toxicity against normal hepatocytes. These results strongly suggest that the F7 fraction of Moroccan cobra *Naja haje* venom obtained by gel filtration chromatography possesses the ability to inhibit cancer cells proliferation. More research is needed to identify the specific molecule(s) responsible for the anticancer effect and investigate their mechanism of action.

## 1. Introduction

More than 745,000 deaths worldwide were directly caused by hepatocellular carcinoma (HCC) in 2012 [1]. It is the most common primary malignancy, currently the sixth most common cancer worldwide, the second deadliest cancer for men and sixth for women [2]. HCC represents the major form of liver cancer; several factors are involved in the development of this cancer, notably chronic infection with hepatitis B (HBV) or hepatitis C (HCV). Numerous other factors such as autoimmune hepatitis, high alcohol consumption and other metabolic diseases such as diabetes and obesity are also risk factors [3,4]. Tumor progression, metastasis and a high frequency of tumor recurrence are still the main cause of death [5]. Cancer cells differ from normal cells as they have the capacity to bypass the cell cycle checkpoints, which are responsible for maintaining intracellular balance [6]. Hallmarks of cancer include sustaining proliferative signaling, evading growth suppressors, resisting cell death, enabling replicative immortality, inducing angiogenesis, and activating invasion and metastasis. In addition, there are two emerging characteristics, notably deregulating cellular energetics and avoiding immune destruction [7]. The development of a therapy of choice with high potency and effectiveness has led to an increased use of anticancer drugs produced by natural resources [8].

Therefore, there is an urgent need to develop new molecules to overcome this problem. To the best of our knowledge, sorafenib is currently the only systemic drug adopted for the treatment of advanced cases of HCC, in addition to other medical and surgical treatment options [9,10]. Liver resection and liver transplantation are not efficient treatments for patients with HCC due to the advanced stage of the disease at the time of diagnosis [11]. There has been a high level of interest in the establishment of natural products for therapeutic purposes. The search for new molecules could be used as antitumor agents with less severe side effects than the usual chemotherapy, and could serve as molecular models for the development of anticancer drugs [12,13].

Over the years, animal venoms and toxins from several species such as snakes, scorpions, cone snails, bees and wasps have been widely studied for their potential as a major source of bioactive molecules [14]. Animal venom has long been considered as a natural therapeutic source. Over time, scorpions have developed a variety of proteins within their venom gland for predator defense as well as to aid in the digestion of prey. This venom is a mixture of salts, nucleotides, lipids, neurotoxins, peptides and proteins. Toxins are the most studied proteins for their neurotoxic and pharmacological activities on ion channels [15]. They are disulfide-bridged peptides (DBPs), which are classified according to their specific target channels into four major groups—the toxin interacting/targeting the Na+, K+, Ca++ and Cl− channels [16]. They are commonly known for their deleterious effects on cells and some of them have important activities for the development of antimicrobial, antimalarial, immunosuppressive and anticancer drugs [17]. Many toxins isolated from snake venom have been used as medical tools to understand different pathophysiological effects, since these proteins have diverse biological effects, such as antiparasitic, antimicrobial and antitumor activities [18,19]. 

Snake venom has been applied for thousands of years in different communities such as China and Africa. Substantial in vivo and in vitro evidence has shed light on the ability of scorpion venom to decrease cancer growth, inhibit cancer progression and metastasis. The anticancer activity of snake venom is based on the immunosuppressive, cytotoxic and antiproliferative power of their proteins, which induce the apoptosis of cancer cells [20]. Recently, venoms have been used in medicine for treating a variety of disease [21]. Several studies have shown a potential anticancer effect of peptides extracted from animal venom. Therefore, the use of peptides from snake and scorpion venoms is one of the therapeutic methods providing very relevant results in the field of fight against cancer [22,23]. Recently, the 3D cell culture model has been widely used in order to create an in vitro microenvironment of cancer similar to that in vivo. This culture technique allows us to test the efficacy as well as the specificity of the compounds against the HCC.

In our study, we studied the potential anti-cancer effect of Moroccan cobra *Naja haje* venom and its fractions purified by gel filtration chromatography on MCTS-based phenomic screening system. The multicellular tumor spheroid model was used by combining HCC cell line Huh7.5 together with WI38 human fibroblast cell, LX2 human hepatic stellate cell line, and human endothelial cell (HUVECs). Moreover, we have studied the effect of venom and its fraction on the normal hepatocytes cell line (Fa2N-4 cell) in order to determine their toxicity and their specificity against the hepatocellular carcinoma in 3D cell culture. The fraction of venom that has an anti-cancerous effect against HCC has been characterized by liquid chromatography coupled with tandem mass spectrometry to determine the molecules responsible for the anticancer effect.

## 2. Results

### 2.1. Fractionation of Venom by Gel Filtration Chromatography

**Figure 1:**
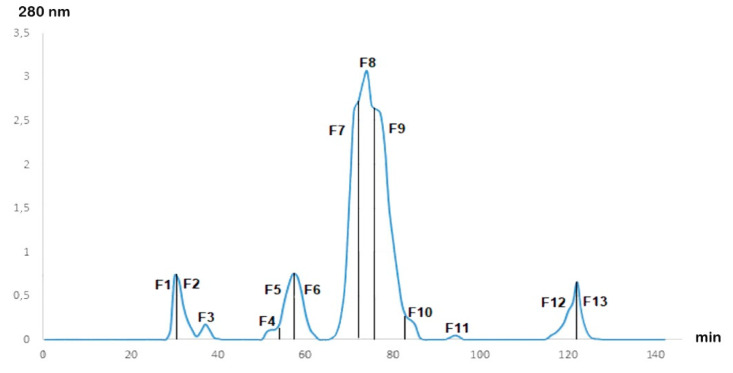
Gel filtration chromatogram of *Naja haje* venom.

After fractionation of the Cobra *Naja haje* venom by gel filtration chromatography, the chromatogram (Figure 1) showed the presence of 13 fractions at different concentrations. The 13 fractions were separated by their molecular weight. F7, F8 and F9 were the major fractions present in *Naja haje* venom. 

### 2.2. Cytotoxicity Test of Venoms and Its Fractions to Normal Hepatocytes

**Figure 2:**
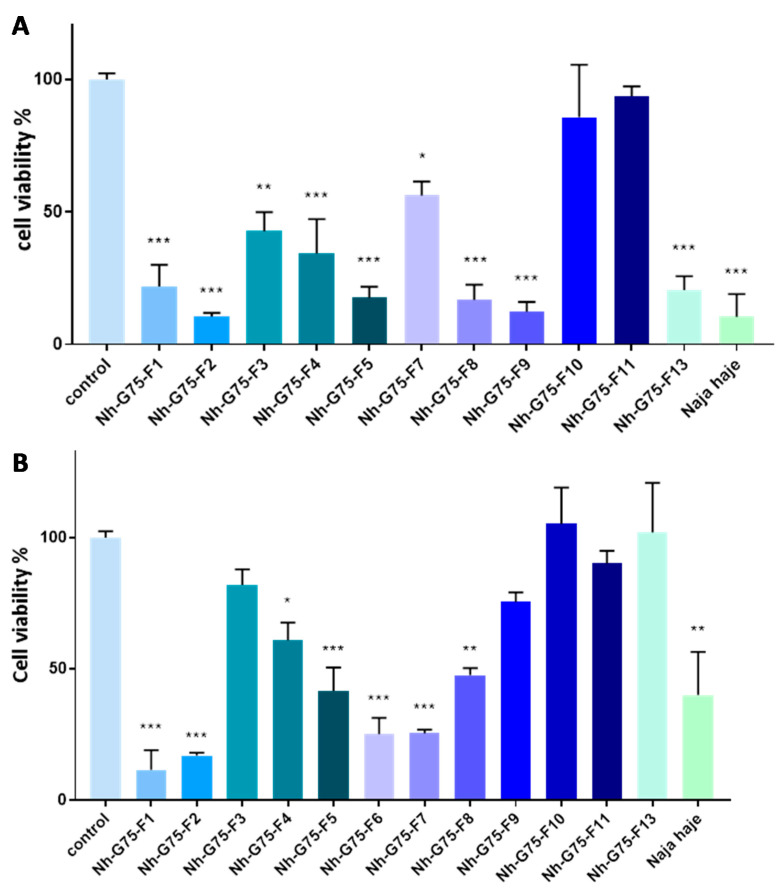
Cytotoxic effect of *Naja haje* venom and its fractions (F1, F2, F3, F4, F5, F6, F7, F8, F9, F10, F11 and F13) and control (PBS) on normal hepatocytes (Fa2N-4). (**A**) Concentration of 10 µg/mL. (**B**) Concentration of 50 µg/mL. Each value is expressed as mean ± standard deviation (*n* = 2). * *p* ≤ 0.05 ** *p* ≤ 0.01 *** *p* ≤ 0.001.

Figure 2A showed cytotoxicity of the venom of *Naja haje* and its fraction at 10 µg/mL on normal hepatocytes (Fa2N-4 cell). F3, F9, F10, F11 and F13 demonstrated a cytotoxic effect in comparison with the negative control (PBS). F4, F8 and the crude venom showed a significant cytotoxic moderated effect; F1, F2, F5, F6 and F7 demonstrated a significant high toxic effect on Fa2N-4 cell. 

The cytotoxic effect of the *Naja haje* venom samples were tested on normal hepatocytes (Fa2N-4) at 50 µg/mL (Figure 2B). F10 and F11 showed a non-significant cytotoxic effect in comparison with the negative control. F3 and F7 showed a moderated cytotoxicity effect. However, it is important to note that F1, F2, F4, F5, F8, F9, F13 and the crude venom demonstrated a high toxic effect on the Fa2N-4 cell line at 50 µg/mL.

### 2.3. Anticancer Activity of Venoms and Its Fractions against MCTS

**Figure 3:**
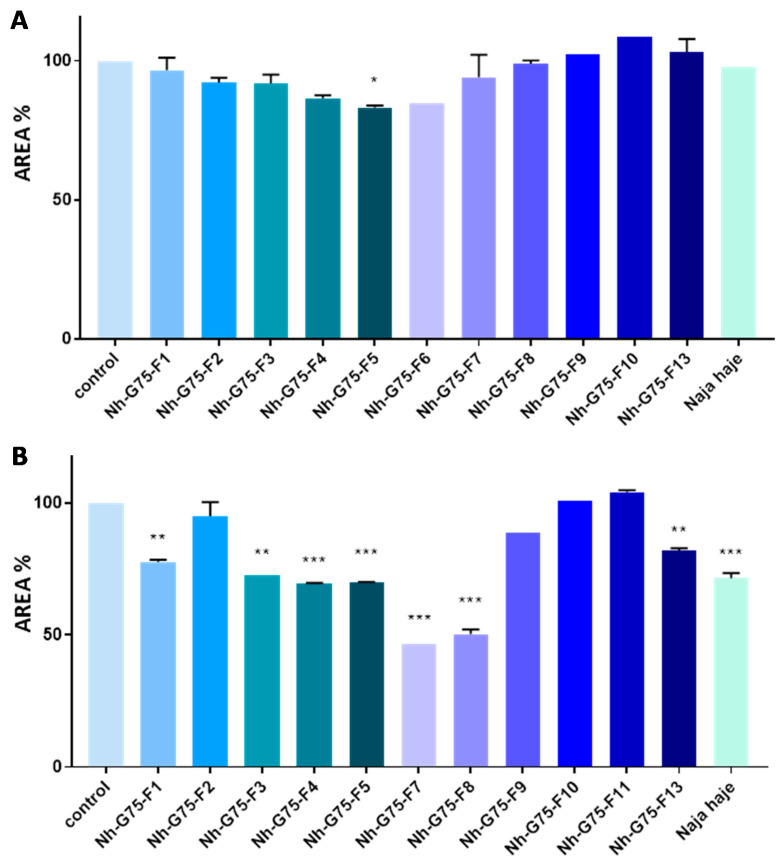
Anticancer effect of *Naja haje* venom and its fractions (F1, F2, F3, F4, F5, F6, F7, F8, F9, F10, F11 and F13) and control (PBS) on the size of the MCTSs. (**A**) Concentration of 10 µg/mL. (**B**) Concentration of 50 µg/mL. Each value is expressed as mean ± standard deviation (*n* = 2). * *p* ≤ 0.05 ** *p* ≤ 0.01 *** *p* ≤ 0.001.

*Naja haje* venom and its fraction were evaluated against MCTSs at 10 µg/mL. All fractions did not show any reduction in the MCTSs area. However, only the F5 fraction showed a low reduction in the MCTSs area in comparison with the negative control (MCTSs treated with PBS) (Figure 3A). 

*Naja haje* venom and its fraction were evaluated against MCTSs at 50 µg/mL. F2, F9, F10 and F11 did not show any effect on the MCTSs area. F1, F3 and F13, however, showed a moderated decrease of the MCTSs area in comparison with the negative control. F4, F5, F7, F8 and the crude venom showed a significant inhibition of MCTSs cells proliferation then a diminution of spheroids size (Figure 3B). 

**Figure 4:**
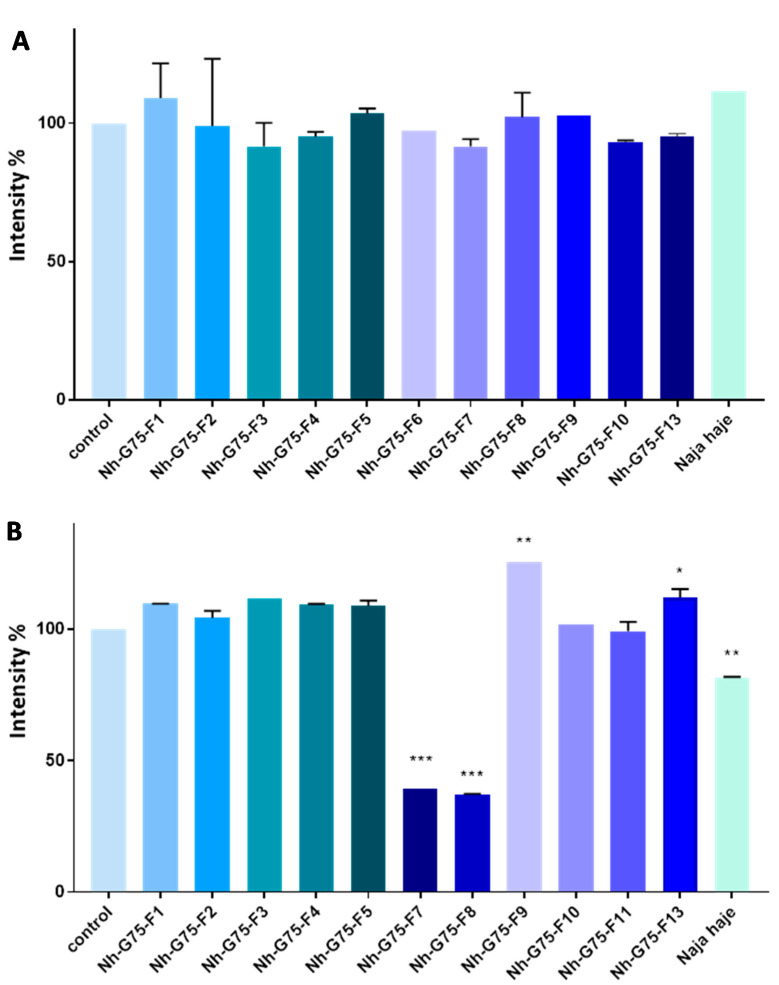
Anti-cancer effect of snake *Naja haje* venom and its fractions (F1, F2, F3, F4, F5, F6, F7, F8, F9, F10, F11 and F13) and control (PBS) on the intensity of RFP of the MCTSs. (**A**) Concentration of 10 µg/mL. (**B**) Concentration of 50 µg/mL. Each value is expressed as mean ± standard deviation (*n* = 2). * *p* ≤ 0.05 ** *p* ≤ 0.01 *** *p* ≤ 0.001.

RFP imaging showed that after treatment with 10 µg/mL of Moroccan cobra *Naja haje* venom and its fractions, no significant reduction in the intensity was observed (Figure 4A). 

Figure 4A shows MCTSs-RFP intensity of cobra *Naja haje* venom and its fractions treatment at 50 µg/mL. F1, F2, F3, F4, F5, F10 and F11 did not enhance any effect on MCTS intensity, while F9 and F13 raised it. Crude cobra venom and both F7 and F8 showed low intensity in comparison with the negative control (PBS).

**Figure 5:**
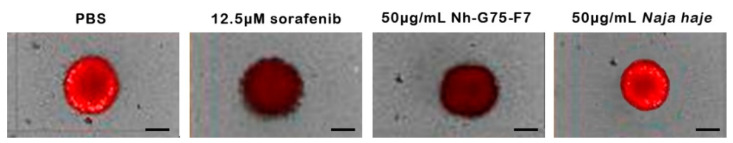
MCTSs towards sorafenib and fraction Nh-G75-F7 (PBS, 12.5 µM sorafenib, 50 μg/mL Nh-G75-F7, 50 μg/mL *Naja haje* crude venom). Scale bar = 200 μm.

Figure 5 showed the appearance of the spheroids to show the ability of the F7 fraction which is similar to sorafenib. The positive and negative controls were 12.5 µM sorafenib and PBS, respectively. Spheroids treated by sorafenib and Nh-G75-F7 showed a decrease in the intensity of RFP, while *Naja haje* crude extract showed similar intensity to the negative control. 

### 2.4. Dose Response Curve of the Nh-G75-F7 Fraction

**Figure 6:**
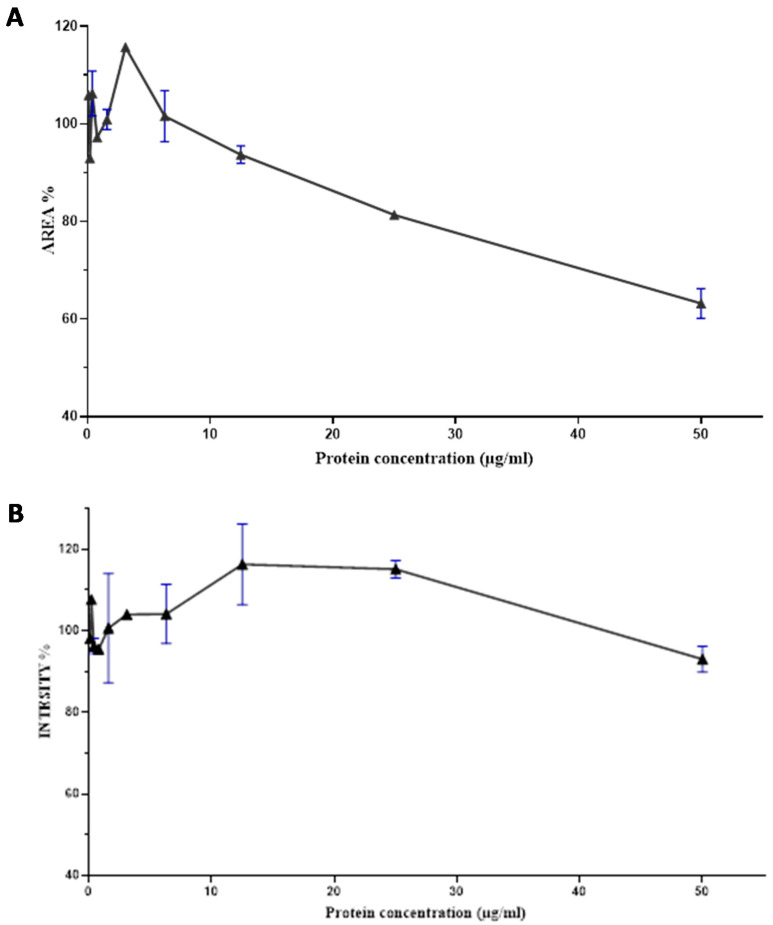
Dose–response curve of the effect of the Nh-G75-F7 fraction. (**A**) AREA of MCTSs. (**B**) RFP intensity of MCTSs.

The F7 fraction of cobra *Naja haje* venom showed that the higher the concentration of proteins, the more the size of spheroids decreases. Thus, an increase in the dose causes a decrease in area spheroids and its toxic effect. This is the dose–effect relationship (Figure 6A). 

According to Figure 6B, there is a correlation between the dose and the effect generated by the F7 fraction of cobra *Naja haje* venom; that being said, an increase in the dose leads to an increase in RFP intensity up to 15ug/mL and then a decrease reaching up to 50 µg/mL.

### 2.5. Molecules Identification in the F7 Fraction of the Naja haje Venom by Mass Spectrometry

**Figure 7:**
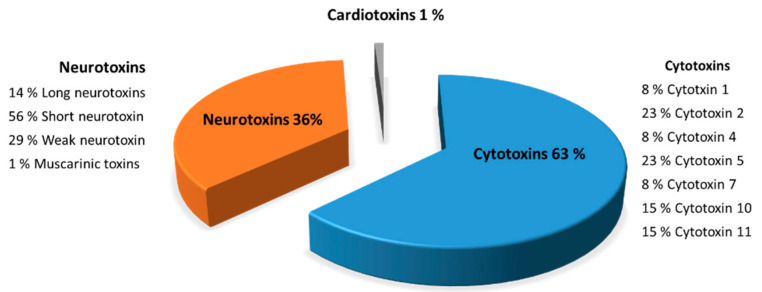
Characterization of the F7 fraction of *Naja haje* venom by mass spectrometry (LC/MS-MS).

Characterization of the toxins from F7 fraction (Figure 7) is described in detail to illustrate the peptides composition from *Naja haje* venom. F7 fraction contains a mixture of cytotoxins, neurotoxins and cardiotoxins, showing 63, 36 and 1%, respectively. Eight peptide cytotoxins—cytotoxin 1 (8%), cytotoxin 2 (23%), cytotoxin 4 (8%), cytotoxin 5 (23%), cytotoxin 7 (8%), cytotoxin 10 (15%) and cytotoxin 11 (15%)—were identified in F7 fraction from the *Naja haje* venom. Moreover, the *Naja haje* venom possesses neurotoxins such as muscarinic toxins, long neurotoxins, weak neurotoxins and the predominate one—the so-called “short neurotoxins”—and constitute 56%. That being said, it is clear that F7 fraction from the *Naje haje* venom contains a number of toxins that may contribute to venom toxicity toward cancer cells.

## 3. Discussion

Treatment options for HCC are divided into surgical resection of the tumor, and the treatment with the multi-kinase inhibitors such as sorafenib; however, it showed only partial clinical efficacy in patients with liver cancer [24,25]. Moreover, it was established that HCC rapidly becomes sorafenib-resistant [26]. To improve treatment options, the discovery of new drugs for HCC is necessary to improve the clinical treatment of those patients.

Venoms have attracted the attention of researchers engaged in the identification of active components and the development of new drug candidates because of their high sensitivity and specificity for target molecules. They have been used in traditional medicine, mainly in Asia and Africa. Cobra venom has been used to treat joint pain, inflammation and arthritis in traditional Indian medicine [20]. Several studies have shown the remission of tumor cells after treatment with molecules derived from venoms [27]. The potential of anti-cancer therapy based on animal venom proteins and peptides from (scorpions, snakes, bees, spiders and toads) has been studied for decades [28].

This present work focuses on studying the anticancer effect of the Moroccan cobra *Naja haje* venom and its fractions purified by gel filtration chromatography against hepatocellular carcinoma using the MCTS model. This 3D culture allowed us to mimic the tumor microenvironment by cultivating cancer cell line Huh 7.5 with WI38 human fibroblast cell, LX2 human hepatic stellate cell line, and human endothelial cell (HUVEC). The toxicity of venom and these fractions against normal hepatocytes was tested on the Fa2N-4 cell line. 

Our results showed that F7 and F8 fractions of gel filtration chromatography at a dose of 50 μg/mL exhibited an anti-HCC effect. F7 and F8 fractions significantly reduced the size of HCC cell line-derived spheroids by decreasing the RFP signal intensity related to cell proliferation. F8 fraction showed significant cytotoxicity towards normal hepatocytes at 50 μg/mL. On the other hand, F7 fraction showed low cytotoxicity. The results of the dose–response curve showed that F7 fraction has a dose-dependent anti-HCC effect.

A previous study has shown the antitumor activity of *Naja haje* venom (NHV) and its fractions (NHVI, NHV-Ia, NHV-Ib, NHV-Ic, NHV-II, NHV-III, and NHV-IV) against several human cell lines, namely lymphoblastic leukemia (1301) cell, hepatocellular carcinoma (Hep-G2), colon carcinoma (HCT-116), cervical carcinoma (HeLa), histiocytic lymphoma, and breast adenocarcinoma (MCF-7). This study revealed that NHV was highly cytotoxic to Hep-G2 and 1301 cells with an IC_50_ values of 6.52 and 4.74 µg/mL, respectively. Additionally, NHV-I fraction, unlike the others, possessed a potential cytotoxic effect against Hep-G2, HeLa, and 1301 cells [29].

Snake venoms in the Elapidae family have been shown to have a significant anti-tumor activity [30]. The crude venom of *Naja naja oxiana* is a powerful inducer of apoptosis in the cancer cell line, including HCC, with minimal side effects on normal cells. A study on the venom toxins of *Naja naja oxiana* showed that the treatment of HepG2 cells with these toxins at concentrations of 15 µg/mL induced cell cycle arrest, and both types of cell death—apoptosis and necrosis—were observed [31]. The crude venom of *Naja naja oxiana* induced an increase in the level of reactive oxygen species (ROS) via the disruption in the mitochondrial of the liver of HCC rats. This process resulted in a drop in matrix metalloproteases (MMP), impaired mitochondrial swelling and release of cytochrome c, which can induce the initiation of the apoptosis signaling pathway by activation of caspase-3 in HCC rats [32]. The Caspian cobra venom *Naja naja oxiana* has revealed that cytotoxins I and II can easily penetrate into living cancer cells and accumulate in lysosomes, which suggest that lysosomal damage is the cause of cell death induced by these two toxins [33]. BthTX-I is a myotoxin isolated from the venom of the viper *Bothrops jararacussu*; this myotoxin revealed cytotoxic activity for the human HepG2 cell line by inducing cell death by apoptosis. BthTX-I may be able to promote the delay in the G0/G1 phase of the cell cycle of murine tumor cells [34]. The viper venom *Echis Pyramidum* has shown a reduction in a dose-dependent cell viability against the HepG2 cell line, by induction of morphological changes and an apoptotic profile compared to an untreated cell control [22]. Venom from Medusa *Nemopilema nomurai* inhibits the proliferation of HepG2 cells at doses (0.8 to 1.2 µg/mL) by induction of apoptotic cell death, while showing no toxicity to hepatocyte, fibroblasts and keratinocytes at the same concentrations. They did not observe any toxic side effects liver and heart tissue, such as changes in the level of enzymes linked to function or histological abnormalities in vivo [35]. *Macrothele raven* venom can inhibit cells invasion and metastasis in the subrenal capsule xenograft model of liver cancer in a dose-dependent manner; its antitumor activity seems to be related to the inhibiting of the signaling PI3K-Akt-mTOR and increase its expression [36]. A study on the venom of another spider called *Haplopelma hainanum* exhibited potent inhibition effects in HepG2 cell proliferation, through reducing the potential of the mitochondrial membrane, caspase-3 and 9 activation, and inducing apoptosis by a pathway dependent on mitochondria [37].

The characterization of gel filtration chromatography F7 fraction of *Naja haje* venom by Nano-LC-MS/MS showed that this fraction is composed of cytotoxins, neurotoxins and cardiotoxins, showing 63, 36 and 1%, respectively. A presidential study on Moroccan cobra *Naja haje* venom revealed that this venom is a very complex mixture that has a total of 76 proteins identified from the database that can be assigned into nine protein families, short neurotoxins, long neurotoxins, weak neurotoxins, neurotoxin-like proteins, muscarinic toxins, cardiotoxins, cytotoxins, cobra venom factor (CVF), L-amino-acid oxidases (LAAO), acetylcholinesterase (AChE), snake venom metalloproteinases (SVMP), cysteine rich secretory proteins (CRISP), venom nerve growth factor (vNGF), phospholipases A2 (PLA2), vespryns, and kunitz-type inhibitor [38]. The anticancer effect of F7 fraction is probably due to the bioactive peptides found in the toxic *Naja haje* venom that may act by inhibiting specific ion channels related to HCC cancer—for example, blocking the voltage-gated shaker potassium channel that was found to mediate tumor cell proliferation by binding to HERG [39], a potassium channel protein that increases in concentration on the cell surface of cancer cells. Moreover, those bioactive venoms may act by activating caspase 9 and 3 and inducing poly (ADP-ribose) polymerase (PARP) cleavage that may deplete the stores of cellular NAD^+^ and induce a progressive ATP depletion, and thus, apoptotic cell death [40]. Several studies have shown many novel modes of anti-cancer mechanism beyond venom peptides in membrane pore formation. For further study, it is necessary to elucidate the mechanism of anticancer effects depending on the sequence or structure of venom peptides [41].

This article highlights the possible therapeutic option for *Naja haje* venom and its fractions on HCC. However, there is still a long way to go before these venom fractions can be successfully used as therapeutic agents against HCC as several clinical trials have to be made for the drug product immunotoxicity. Moreover, purification of the active fraction is needed to identify the molecule responsible for anti-HCC activity.

## 4. Conclusions

This work shows that the snake *Naja haje* venom, specifically the F7 fraction from the gel filtration chromatograph, showed a dose-dependent effect on the reduction of the size of the HCC-MCTSs, and thus the decrease of RFP intensity of the cells of the cancerous line, indicating the cytotoxic effect of this fraction against HCCs. In this regard, we propose that it is necessary to continue our research and carry out a refraction by filtration gel chromatography of the F7 fraction from cobra *Naja haje* venom in order to elucidate the effect of each fraction obtained by HPLC and subsequently identify the molecule(s) responsible for cytotoxicity against HCC cells.

## 5. Materials and Methods

### 5.1. Cell lines and Culture Conditions

The HCC cell line Huh7.5-red fluorescent protein-(RFP)-NLS-IPS reporter cell line was kindly provided by Dr. Mark Windisch (Institut Pasteur Korea, Gyeonggi-do, Korea). Stromal cells WI38 (human fibroblasts), LX2 (human hepatic stellate cells), and HUVECs (human umbilical vein endothelial cells) were obtained from ATCC (Manassas, VA, USA). Fa2N-4 cells (an immortalized normal hepatocyte cell line) were obtained from Xenotech (Lenexa, KS, USA). 

Huh7.5 cell line were cultured in Dulbecco’s modified Eagle’s medium (DMEM; Welgene) supplemented with 10% fetal bovine serum and 1% penicillin/streptomycin. WI38 cells were cultured in minimum essential media (MEM; Welgene, Daegu, Korea) supplemented with 10% heat-inactivated fetal bovine serum (FBS; Gibco, Grand Island, NY, USA) and 1× penicillin-streptomycin (P/S; Gibco). LX2 cells were cultured in DMEM supplemented with 10% heat-inactivated FBS and 1× P/S. HUVECs were cultured in endothelial basal medium (EBM) obtained from Promo Cells. All cell lines were maintained at 37 °C in a humidified atmosphere of 5% CO_2_.

Fa2N-4 cells were plated to the flask with serum-containing plating medium (XenoTech, Lenexa, KS, USA). After cell attachment (3 to 6 h), plating medium was replaced with MFE serum free supporting (SF) medium (XenoTech), which is a nutrient-rich medium for maintaining Fa2N-4 cells in culture.

### 5.2. Snake venom

The venom of *Naja haje* was obtained by manual stimulation of the Cobra venom gland kept in captivity at the Serpentarium of the animal unit at the Pasteur Institute of Morocco. The pooled venom was centrifuged at 15,000× *g* for 15 min at 4 °C to remove debris, before lyophilization and storage at −20 °C until use [42].

### 5.3. Fractionation of Venom by Gel Filtration Chromatography

*Naja haje* venom (161 mg) was fractionated in a glass column (2.6 × 100 cm; Pharmacia, Uppsala, Sweden) with a Sephadex G75 Medium gel (Sigma Aldrich, Lyon, France) and equilibrated with 10% acetic acid. A continuous flow rate of 26 mL/h is provided by a Pump P-1 peristaltic pump (Amersham Biosciences, Piscataway, NJ, USA). The collection was carried out by a Frac-920 automatic fraction collector (Amersham Biosciences, Piscataway, NJ, USA) at the rate of 2.5 mL in each tube [43].

### 5.4. Cytotoxicity Test of Venoms and Its Fractions to Normal Hepatocytes (Fa5N4)

Fa2N-4 cells were seeded at a density of 3.5 × 10^3^ cells/well in 384-well plates. After 24 h of incubation, crude venom and its fractions were resolubilized in phosphate-buffered saline (PBS; Lonza) at a concentration of 10 and 50 µg/mL, then added and incubated for 48 h. A solution of PBS was used as a negative control and 12.5 µM sorafenib (Santa Cruz Biotechnology) as a positive control. Thus, cells were fixed with 4% PFA for 10 min at room temperature and washed twice with DPBS. Hoechst 33342 was used for nuclear staining. To capture enough cells (>1000) for analysis, five image fields were collected from each well, starting at the center of the well. All of the image analysis was acquired with an Operetta high-content imaging system and Harmony software. Cell counts were calculated and normalized to the control (PBS) [26].

### 5.5. Anticancer Activity of Venoms and Its Fractions against MCTS (Multicellular Tumor Spheroid)

Single cells were suspended at 6 × 10^3^ cells/well (384-well ultra-low attachment round-bottom ULA microplates (Greiner Bio-One, Monroe, NC, USA) in DMEM media at 37 °C for three days. To produce MCTSs, four kinds of cells (RFP-Huh7.5 cells, LX2 cells, WI38 cells, and HUVECs) suspended in DMEM media were seeded at a density of 6 × 10^3^ cells/well at 37 °C for three days. Crude venom and its fractions were resolubilized in phosphate-buffered saline (PBS; Lonza) at a concentration of 10 and 50 µg/mL, then added and incubated for an additional seven days. A solution of PBS was used as a negative control and 12.5 µM sorafenib (Santa Cruz Biotechnology) as a positive control. All of the image, area and intensity analysis of spheroid was performed using the HCS system and Harmony software. The criteria of spheroid area were calculated by average of positive control and negative control. RFP-intensity criteria were calculated by difference of average and standard deviation of negative control [26].

### 5.6. Dose Response Curve

Fractions that showed anti-cancerous activity with low cytotoxicity were tested at different concentrations (50, 25, 12.5, 6.3, 3.1, 1.6, 0.8, 0.4, 0.2 and 0.1 μg/mL) in 3D culture in order to establish the dose–response curve of these fractions against spheroids of the hepatocellular carcinoma by the same protocol on MCTSs. Sorafenib was tested as a positive control at different concentrations (starting from 100 µM, 10 points, twofold serial dilution) [26].

### 5.7. Molecules Identification in the F7 Fraction of the Naja haje Venom Nano-Liquid Chromatography Coupled to Tandem Mass Spectrometry (Nano-LC-MS/MS)

#### 5.7.1. Reduction, Alkylation and Trypsin Digestion

The lyophilized *Naja haje* fraction was suspended in 10 µL of 4 M urea in 100 mM NH_4_HCO_3_ (*v*:*v*), then mixed with 10 mM of freshly prepared dithiothreitol (DTT) in 100 mM NH_4_HCO_3_ (*v*:*v*). The mixture was sonicated and flushed with nitrogen before being heated for two hours at 60 °C. The free sulfhydryl groups of cysteine were alkylated by adding 55 mM of iodoacetamide (IAA) in 100 mM NH_4_HCO_3_ (*v*:*v*), the samples were incubated at room temperature for 20 min, then 5 µl of 30 nM DTT were added and incubated over one hour to remove the excess of IAA. Subsequently, the reduced and alkylated fraction was digested overnight at 37 °C, with 1 µg of sequencing Grade Modified Trypsin (Promega) in 50 mM NH_4_HCO_3_. The enzymatic reaction was quenched by adding 5 µL of formic acid (FA) 5%. Then, the digested fraction was dried and resuspended in 0.1% (*v*:*v*) FA and 5% (*v*:*v*) acetonitrile (ACN) [44].

#### 5.7.2. Nano-LC-MS/MS Analysis

Proteomic analysis of digested *Naja haje* fraction was carried out in an Agilent 1200 Series HPLC-Chip/MS system connected to a 6520 Quadrupole-Time of Flight (Q-TOF) mass spectrometer, equipped with a nano-electrospray source (Agilent Technologies, Santa Clara, CA, USA). For the online fractionation, two microliters of tryptic peptides were loaded and enriched on a 160 nL RP-C18 trap column, then separated on an analytical nano-column (150 mm × 75 µm) packed with ZORBAX SB-C18, 5 µm, 300Å (G4240-62010; Agilent Technologies). The separation was maintained over 25 min at 450 nL/min, using a linear gradient from 3 to 80% ACN in 0.1% FA. The eluted peptides were operated in positive mode in the Q-TOF mass spectrometer; the mass range was set from 290 to 1700 *m*/*z* and from 59 to 1700 for the MS and MS/MS scans, respectively. The total cycle time was two seconds. In each cycle, five of the most abundant precursor ions were subjected to MS/MS fragmentation; the ions with single charge were excluded, while the collision energy was automatically adjusted according to *m*/*z* [44].

#### 5.7.3. Data Processing

MS-MS data files were processed using the Peaks 7.5 software (Bioinformatics Solutions Inc, Waterloo, ON, Canada) against the UniProtKB/Swiss-Prot database downloaded in November 2018 from NCBI. Specific parameters were set as follows: to carry out the research, mass tolerance of parents and fragments set at 50 ppm and 0.3 Da, respectively; enzymatic specificity, trypsin; three as the maximum missed cleavages for trypsin; variable modification, oxidation (M), carbamidomethylation, pyro-glu of Q and E, dehydration and amidation; no fixed modification was taken into account; instrument, ESI-Q-TOF; taxonomy, all; database, UniProtKB/Swiss-Prot. The characterized proteins/peptides have been classified into different families on the basis of their function according to the UniProt database (https://www.uniprot.org, accessed on 23 February 2019) [44].

### 5.8. Statistical Analysis

The results were expressed as mean ± standard deviation (S.D.) of minimum two independent experiments in duplicate (*n* = 2). 

The data analyses were performed in GraphPad for statistical analysis. To compare the different effects of the fractions, statistical analysis will be assessed with a one-way ANOVA test followed by Dunnett’s multiple comparisons test for parametric data.

## Data Availability

The data that support the findings of this study are available from the corresponding author upon reasonable request.
